# Digital occlusal force analysis in anterior crossbite

**DOI:** 10.1038/s41598-025-16194-z

**Published:** 2025-08-19

**Authors:** Hazal Özer Ünal, Merve Abaklı İnci, Merve Taşcı Dedeoğlu, Ümran Akgül

**Affiliations:** 1 Department of Pediatric Dentistry, Faculty of Dentistry, Necmettin Erbakan University, Yaka Mahallesi Bağlarbaşı Sokak, Meram, Konya, 42090 Türkiye; 2 İskenderun Oral and Dental Health Center, Altınçay, Mehmet Kafadar Cad., Altınçay Mahallesi 623. Sk. No:4, 624. Sk No: 20, Antakya, 31040 Hatay Türkiye

**Keywords:** Anterior crossbite, Occlusal force distribution, Periodontal health, Digital occlusal analysis, Dentistry, Occlusion, Paediatric dentistry, Preventive dentistry

## Abstract

Anterior crossbite is a common malocclusion in pediatric patients that can negatively affect occlusal force distribution and periodontal health. This study aimed to evaluate changes in occlusal force distribution and periodontal health parameters before and after interceptive orthodontic treatment using a digital occlusal analysis device. Thirty-five children aged 6–11 years with anterior crossbite were treated using a Hawley appliance with a Z-spring. Occlusal forces were recorded at baseline (T0) and after treatment (T1) using the OccluSense digital analysis system. Periodontal parameters, including plaque index, gingival index, probing depth, and clinical attachment level, were also assessed. Data were analyzed using paired t-tests, Wilcoxon signed-rank tests, and Kruskal–Wallis tests, with the significance level set at *p* < 0.05. Following treatment, the regional force distribution decreased from 65.3% ± 8.2–52.1% ± 6.7% (*p* < 0.001), and the percentage force distribution decreased from 68.7% ± 9.1–54.3% ± 7.2% (*p* < 0.001). The plaque index declined from 1.8 ± 0.6 to 1.2 ± 0.4, gingival index from 1.5 ± 0.5 to 0.9 ± 0.3, probing depth from 3.2 ± 0.8 to 2.7 ± 0.6 mm, and clinical attachment level from 2.8 ± 0.7 to 2.4 ± 0.5 mm (*p* < 0.05). Interceptive treatment with a Z-spring appliance resulted in statistically significant changes in occlusal force distribution and periodontal health parameters. Digital occlusal analysis may serve as a valuable tool for objectively evaluating treatment outcomes in pediatric patients.

**Supplementary Information:**

The online version contains supplementary material available at 10.1038/s41598-025-16194-z.

## Introduction

Occlusion plays a fundamental role in the overall functionality of the maxillofacial system, particularly in mastication, speech, and temporomandibular joint (TMJ) health^1^. Traditionally, occlusal relationships have been evaluated using articulating paper or wax; however, these conventional methods are limited in their ability to capture the timing and magnitude of occlusal contacts^2^. Technological advancements have led to the introduction of digital occlusal analysis systems that allow for dynamic, real-time, and quantifiable assessment of occlusal forces^3^.

Anterior crossbite is a sagittal malocclusion commonly observed in children^4^. If not addressed promptly, it may lead to aesthetic issues, abnormal mandibular development, TMJ dysfunction, and periodontal complications in the lower anterior region^5,6^.

Interceptive orthodontic treatment, initiated during the mixed dentition period, aims to correct such malocclusions early and guide favorable craniofacial development^7^. A widely used appliance for this purpose is the Hawley appliance with a Z-spring, which is designed to move affected teeth labially^8^.

Among the available tools for occlusal assessment, the OccluSense system is notable for its portability, user-friendly interface, and high sensitivity in recording force intensity and distribution^9^. Although systems such as T-Scan have been widely studied^10^, OccluSense has recently gained attention in pediatric dentistry owing to its practical advantages. Nevertheless, studies validating its application in children remain limited^11^.

Additionally, periodontal indicators such as plaque index, gingival index, probing depth, and clinical attachment level are essential for evaluating the impact of orthodontic interventions^12^. Understanding how interceptive treatment affects occlusal function and periodontal status can assist clinicians in planning and monitoring early interventions more effectively.

This study aimed to evaluate changes in occlusal force distribution and periodontal health parameters before and after interceptive orthodontic treatment in pediatric patients with anterior crossbite using the OccluSense digital analysis system.

The null hypothesis was that interceptive treatment using a Z-spring appliance would not result in significant changes in occlusal force distribution or periodontal health outcomes.

## Materials and methods

### Study design and ethical approval

This study was conducted at the Department of Pediatric Dentistry, Faculty of Dentistry, Necmettin Erbakan University, between March and September 2022. Ethical approval was obtained from the university’s ethics committee (approval number: 2022/792), and written informed consent was obtained from the legal guardians of all participants prior to inclusion. Although data were analyzed retrospectively, the study followed a prospective clinical design with predefined protocols and standardized time points. The study adhered to the STROBE (Strengthening the Reporting of Observational Studies in Epidemiology) guidelines for transparent reporting of observational studies. The completed STROBE checklist is provided in the supplementary material. As this was a single-arm, nonrandomized prospective clinical investigation without intervention allocation, it was not registered in a clinical trial registry.

### Participants and eligibility criteria

Thirty-five pediatric patients aged 6–11 years diagnosed with anterior crossbite involving at least one maxillary incisor were included in the study. Inclusion criteria were good general health, cooperative behavior (Frankl score 3 or 4)^13^, a normodivergent vertical growth pattern, and a Class I molar relationship. Exclusion criteria included the presence of systemic diseases, special healthcare needs, TMJ disorders, parafunctional habits, developmental anomalies, significant facial asymmetry, or existing periodontal disease.

### Sample size calculation

The required sample size was calculated using G*Power software (version 3.1.9.7; Heinrich Heine University Düsseldorf, Düsseldorf, Germany), with an estimated effect size of 0.35 based on values commonly reported in similar clinical studies that involve occlusal analysis^21^, α = 0.05, and power = 0.80^14^. Although the minimum required sample was 21, a total of 35 participants were included to account for potential dropouts.

### Orthodontic intervention

**Fig. 1:**
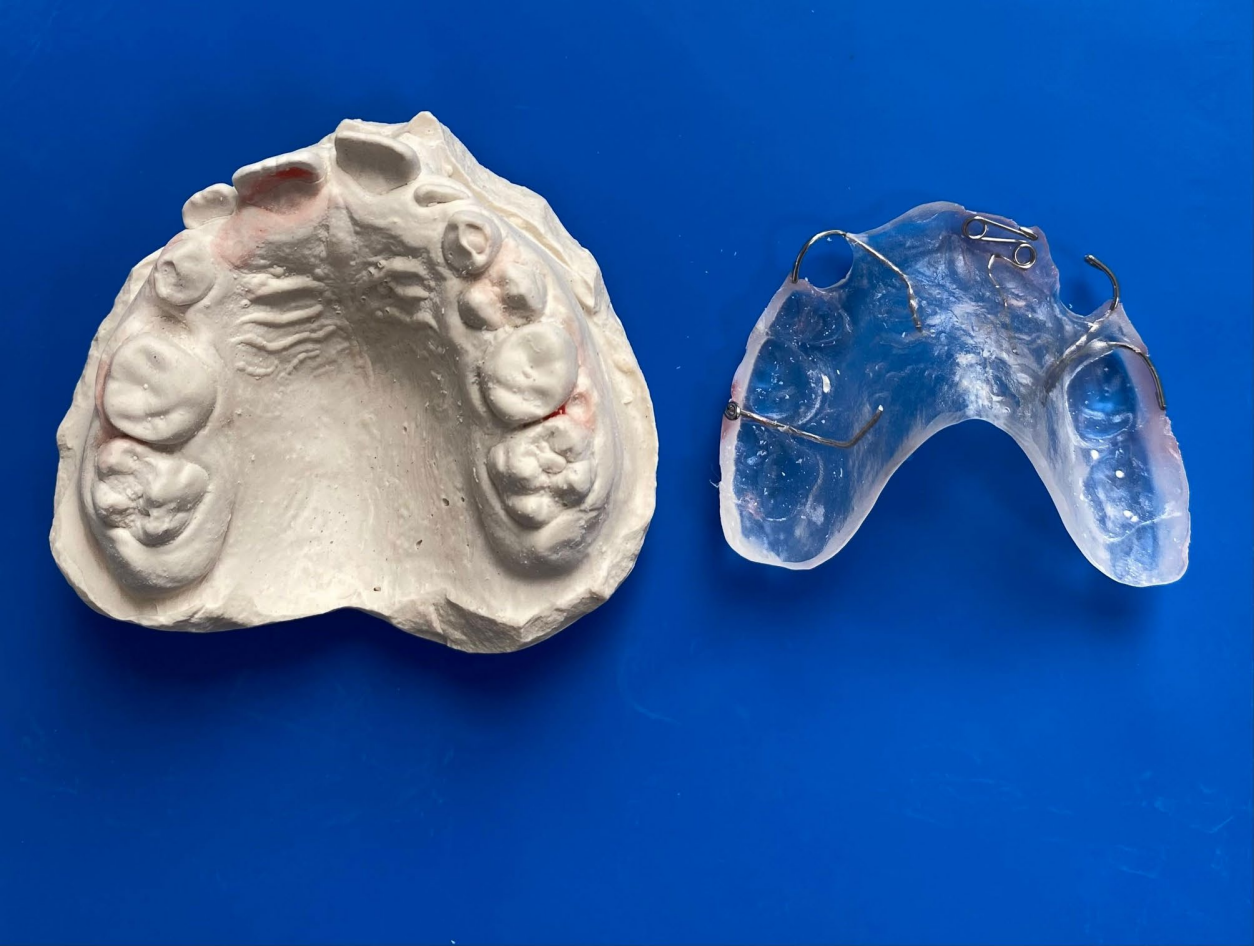
Hawley appliance with a Z-spring.

**Fig. 2:**
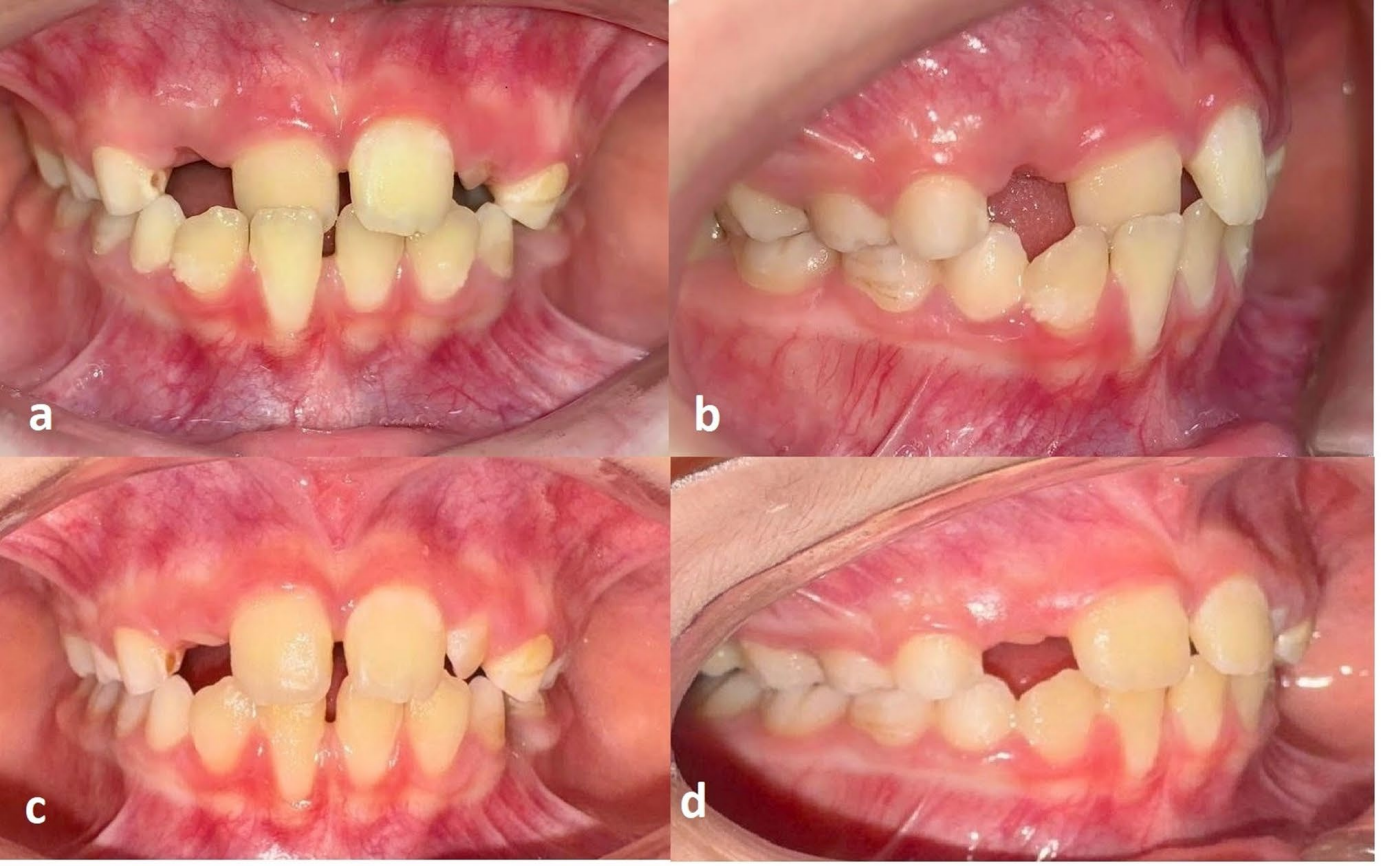
Intraoral views of a representative case showing anterior crossbite involving tooth 11 **(a, b)** Pretreatment frontal and right lateral views demonstrating anterior crossbite of the maxillary right central incisor (tooth 11) **(c, d)** Posttreatment views following interceptive orthodontic treatment with a Z-spring appliance.

All patients underwent interceptive orthodontic treatment using a Hawley appliance fitted with a Z-spring designed to move the affected anterior tooth labially (Fig. 1). Treatment duration ranged from 3 to 17 weeks, with an average duration of 9.95 ± 6.59 weeks. Occlusal and periodontal measurements were obtained at two time points: prior to treatment (T0) and immediately after appliance removal (T1) (Fig. 2).

### Occlusal force assessment

**Fig. 3:**
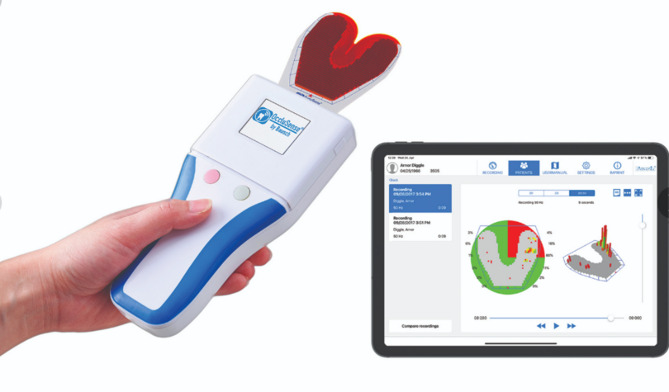
OccluSense digital occlusal analysis device.

Occlusal forces were measured using the OccluSense digital analysis system (Bausch GmbH, Cologne, Germany). The system includes a handheld reader and a 60-µm-thick pressure sensor capable of recording the force across 256 sensitivity levels (Fig. 3). All patients were seated in an upright position and instructed to bite with maximum force in centric occlusion. Device calibration was confirmed with a test sensor before each measurement. Each recording was repeated twice by two independent, trained examiners. Prior to data collection, both examiners were calibrated using a standardized measurement protocol, which included assessing five patients independently and comparing the results using intraclass correlation coefficients (ICCs). An ICC value of > 0.85 was considered acceptable, and calibration was repeated until this threshold was consistently achieved. The average of four values was used for statistical analysis. Regional force distribution (tooth-based) and percentage force distribution (relative to total force) were evaluated.

### Periodontal parameters

Periodontal evaluation included the plaque index (Silness and Löe), gingival index (Löe and Silness), probing depth, and clinical attachment level. All assessments were performed by a single calibrated examiner using a periodontal probe^16^, and data were collected at T0 and T1.

### Statistical analysis

Data analysis was performed using SPSS version 20.0 (IBM Corp., Armonk, NY, USA). The Shapiro–Wilk test was used to assess the normality of data distributions. For normally distributed variables, paired sample t-tests were applied to compare pretreatment and posttreatment values (T0 vs. T1) for occlusal force and periodontal parameters. For non-normally distributed data, the Wilcoxon signed-rank test was used. Group-level effects were assessed using Kruskal–Wallis tests to compare percentage force changes across age groups (6–8 and 9–11 years), sex (male and female), and treatment duration categories (3–6, 7–10, > 10 weeks). No statistically significant differences were observed among these subgroups; thus, post hoc pairwise comparisons were not performed, and no multiple comparison correction (e.g., Bonferroni) was applied. Chi-square tests were used to examine associations between categorical variables. Effect sizes (Cohen’s d) were calculated for the primary outcomes. The effect sizes for the reduction in regional and percentage force distributions were 1.76 and 1.75, respectively, both indicating large and clinically meaningful effects. The level of statistical significance was set at *p* < 0.05. The statistical strategy and subgroup comparisons are summarized in Supplementary Table 1.

## Results

### Participant characteristics

**Fig. 4:**
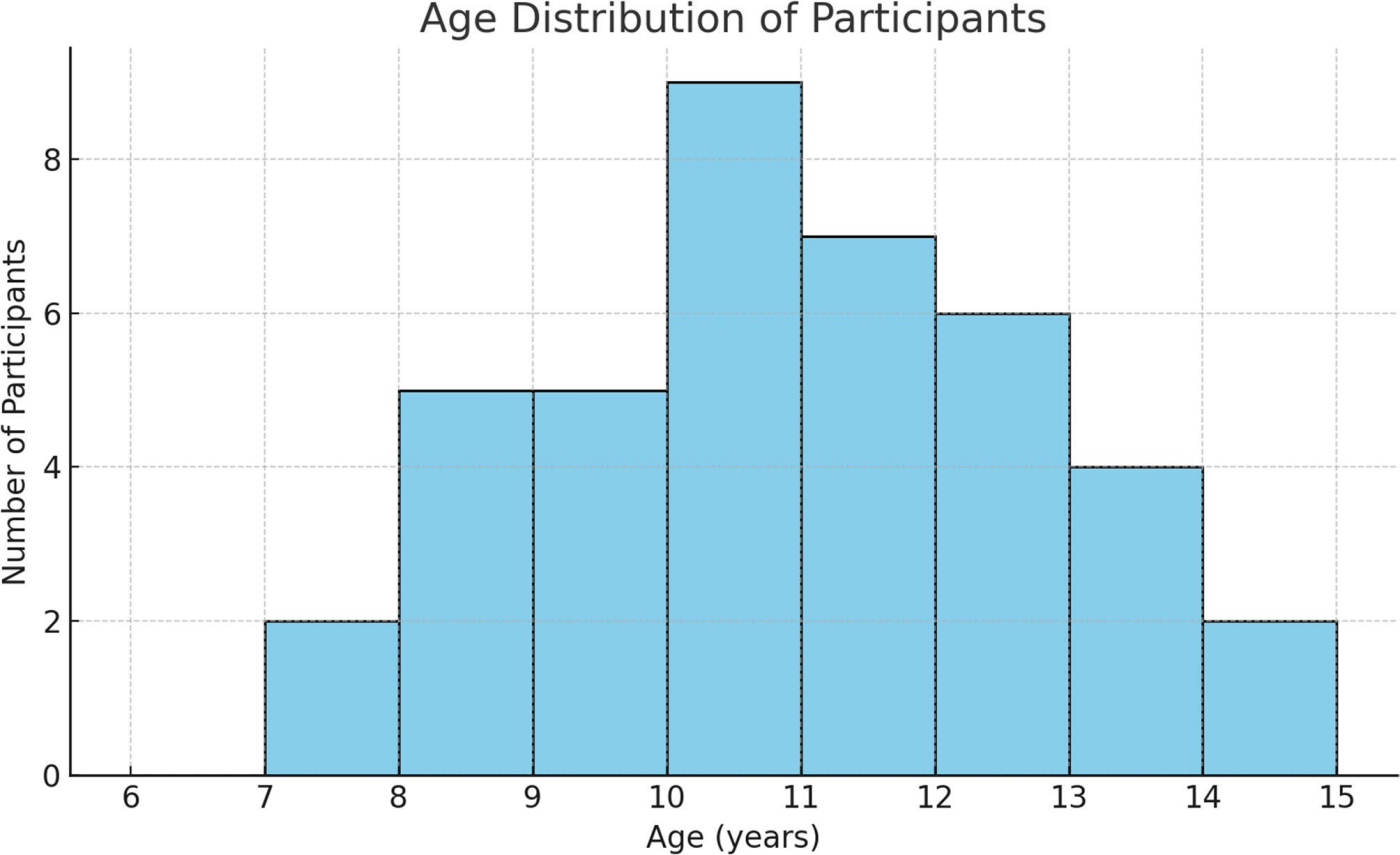
Age distribution of participants.

A total of 35 pediatric patients (23 males and 12 females) aged between 6 and 11 years (mean age: 10.59 ± 1.82 years) were included in the study (Fig. 4). The average treatment duration was 9.95 ± 6.59 weeks (range: 3–17 weeks). No dropouts occurred during the study period.

### Occlusal force distribution

**Fig. 5:**
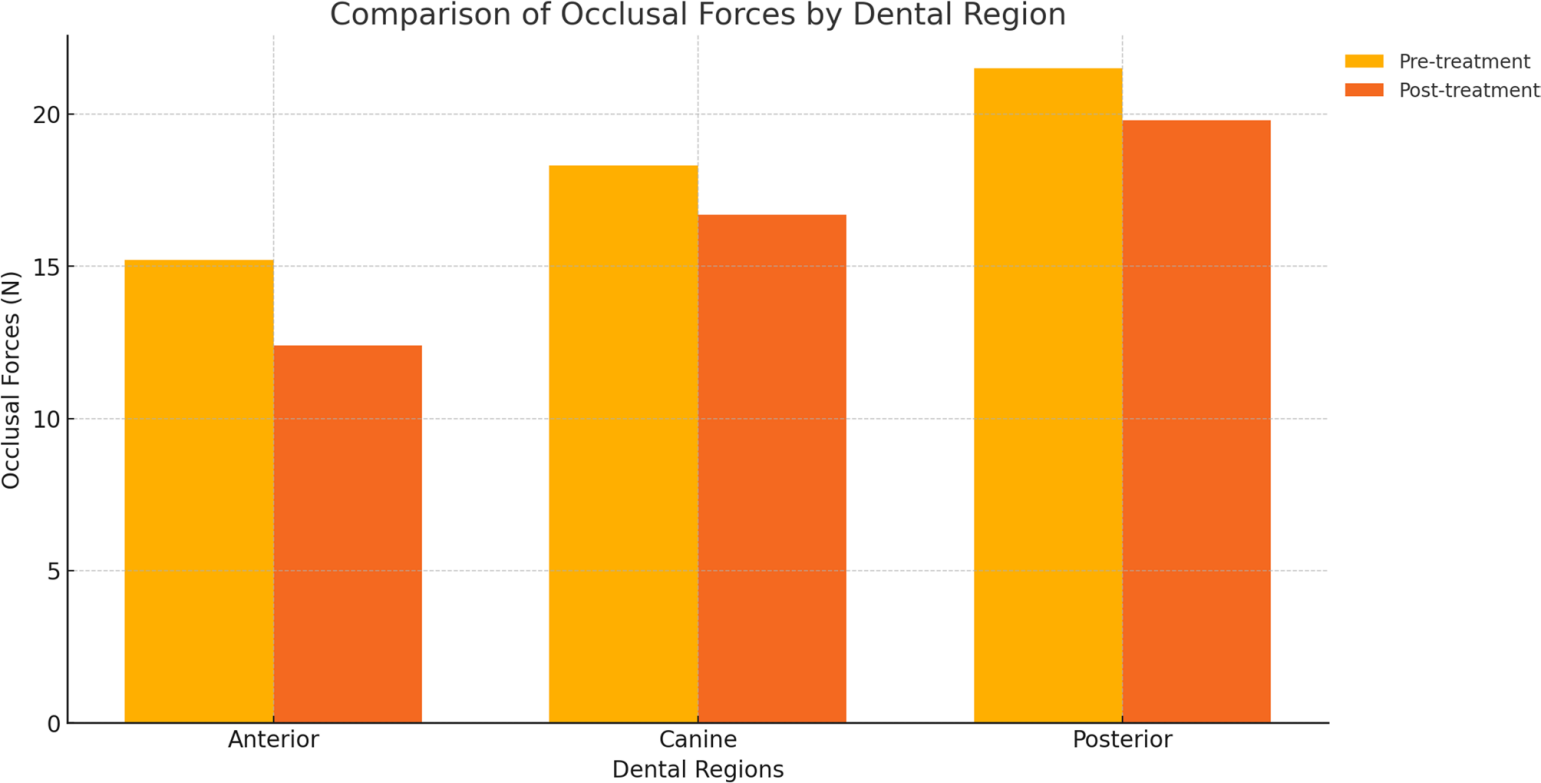
Comparison of occlusal forces before and after treatment by dental region.

A significant reduction in regional and percentage occlusal force distribution was observed following treatment. The mean regional force distribution decreased from 65.3% ± 8.2% at T0 to 52.1% ± 6.7% at T1 (*p* < 0.001, paired t-test), while the percentage force distribution decreased from 68.7% ± 9.1–54.3% ± 7.2% (*p* < 0.001, paired t-test) (Table 1). When analyzed by dental region, occlusal forces declined in all segments: anterior forces decreased from 15.2 to 12.4 N, the canine region from 18.3 to 16.7 N, and the posterior region from 21.5 to 19.8 N (Fig. 5).

**Table 1:** Regional and percentage force distribution before and after treatment (Paired t-test).

Variable	T0 (Mean ± SD)	T1 (Mean ± SD)
Regional Force Distribution (%)	65.3 ± 8.2	52.1 ± 6.7
Percentage Force Distribution (%)	68.7 ± 9.1	54.3 ± 7.2

Data are presented as mean ± standard deviation. Paired t-test was used. *p < 0.001.

### Tooth-Specific force distribution

Among the evaluated teeth, statistically significant reductions in force percentage were observed for the frequently affected maxillary incisors. Specifically, tooth 11 demonstrated a median reduction of 19% (*p* = 0.0001), and tooth 21 showed a 14% median decrease (*p* < 0.0001). Additionally, descriptive observations indicated reductions in force on tooth 14 (from 25 to 20%) and tooth 15 (from 15 to 5%). Although descriptive trends were also noted for teeth 12 and 22, limited sample sizes or nonsignificant test results precluded further statistical interpretation (Table 2). These findings support a general pattern of force redistribution and contribute to the overall interpretation of improved occlusal balance following treatment.

**Table 2:** Tooth-Specific occlusal force distribution (%).

Tooth	T0 (%)	T1 (%)
11	10	15
12	20	25
13	30	35
14	25	20
15	15	5

Data represent percentage force distribution per individual tooth before (T0) and after (T1) treatment.

### Periodontal parameters

All periodontal indices improved significantly after treatment. The plaque index decreased from 1.8 ± 0.6 to 1.2 ± 0.4, the gingival index from 1.5 ± 0.5 to 0.9 ± 0.3, probing depth from 3.2 ± 0.8 to 2.7 ± 0.6 mm, and clinical attachment level from 2.8 ± 0.7 to 2.4 ± 0.5 mm (*p* < 0.05 for all; Wilcoxon signed-rank test) (Table 3). Although effect sizes could not be calculated owing to the use of nonparametric analyses, the magnitude of the observed improvements (mean reductions ranging from 0.4 to 0.9 units) supports their clinical relevance.

**Table 3:** Periodontal index results before and after treatment (Wilcoxon Signed-Rank Test).

Parameter	T0 (Mean ± SD)	T1 (Mean ± SD)	Mean Difference	95% CI	*p*-value
Plaque Index	1.8 ± 0.6	1.2 ± 0.4	0.6	0.35–0.85	< 0.05
Gingival Index	1.5 ± 0.5	0.9 ± 0.3	0.6	0.40–0.80	< 0.05
Probing Depth (mm)	3.2 ± 0.8	2.7 ± 0.6	0.5	0.16–0.84	< 0.05
Clinical Attachment Level (mm)	2.8 ± 0.7	2.4 ± 0.5	0.4	0.10–0.70	< 0.05

Data are presented as mean ± standard deviation. Wilcoxon signed-rank test was applied to evaluate significance.

### Subgroup analysis

No statistically significant associations were found between age group (χ² = 9.65, df = 7, *p* = 0.209), sex (χ² = 0.016, df = 1, *p* = 0.901), or treatment duration group (χ² = 17.5, df = 14, *p* = 0.231) and percentage force changes (Table 4).

**Table 4:** Comparison of percentage force distribution across subgroups (Kruskal-Wallis Test).

Variable	χ²	df	*p*-value
Age Groups	9.65	7	0.209
Sex	0.016	1	0.901
Treatment Duration Groups	17.5	14	0.231

Kruskal-Wallis test was used to assess subgroup differences in percentage force distribution. df: degrees of freedom.

### Total occlusal force range

Although regional force values decreased, total occlusal force increased after treatment. The average total force increased from 25.9 N (range: 19.2–31.3 N) at T0 to 30.4 N (range: 23.2–37.6 N) at T1, suggesting a shift toward more functional and balanced occlusal loading (Table 5). These values are presented descriptively because Newton-based patient-level data were not available in a standardized format suitable for inferential statistical testing. Therefore, the interpretation of total force increase should be considered exploratory.

**Table 5:** Total occlusal force measurements before and after Treatment.

Timepoint	Minimum (*N*)	Q1 (*N*)	Median (*N*)	Q3 (*N*)	Maximum (*N*)
T0	19.2	24.0	26.0	27.5	31.3
T1	23.2	28.0	30.0	32.5	37.6

Occlusal forces measured in Newtons (N). Q1: first quartile, Q3: third quartile.

## Discussion

These findings clearly reject the null hypothesis, which proposed that interceptive treatment with a Z-spring appliance would not result in significant changes in occlusal force distribution or periodontal health outcomes. The observed improvements in both parameters underscore the therapeutic efficacy of early-phase orthodontic intervention, producing measurable clinical effects.

A statistically significant reduction in regional and percentage occlusal force values was observed following treatment, accompanied by a redistribution of load across the dental arch. This shift suggests improved occlusal balance, with functional contacts more evenly distributed and localized mechanical stress reduced. Biomechanically, this pattern aligns with enhanced neuromuscular coordination and occlusal equilibration—frequently cited goals of early orthodontic intervention^4,18^. The simultaneous increase in total occlusal force alongside reductions in segmental values likely reflects a redistribution mechanism rather than a net reduction in force generation. Before treatment, excessive force may have been concentrated in anterior or posterior regions owing to premature contact and skeletal compensation. Following interceptive intervention, occlusal contacts became more evenly distributed, leading to reduced focal stress and broader muscular recruitment. As a result, while segmental peak values declined, the cumulative output from a more harmonized occlusal loading pattern increased. Similar findings have been reported by Yawaka et al.^17^ and Sonnesen & Bakke^19^, supporting the interpretation that occlusal efficiency improves through balanced contact patterns.

The segmental reductions observed in the anterior, canine, and posterior regions suggest that anterior crossbite correction not only repositions teeth but also alleviates abnormal focal loading. By reducing premature incisal and cusp contacts, the treatment facilitates a more physiological load-sharing mechanism during mastication, which can improve functional efficiency and occlusal stability. These findings are consistent with those of Sonnesen and Bakke^19^, who showed that children with crossbite exhibited significantly higher occlusal force intensity in specific segments pretreatment, which normalized after treatment. Similarly, Ge et al.^4^ reported that occlusal adjustments during the mixed dentition phase enhanced neuromuscular coordination and led to more stable occlusal function over time. Yawaka et al.^17^ further demonstrated that correcting anterior occlusal interference in primary dentition resulted in more even force distribution, reinforcing the notion that interceptive orthodontic treatment can positively influence occlusal dynamics even at early developmental stages. Collectively, these studies suggest that our observed segmental force reductions are not due to weakening but rather reflect a redistribution and stabilization of occlusal contacts. Such changes represent a transition from pathological load concentrations to a more efficient, homeostatic masticatory pattern.

The improvements observed in our study are particularly meaningful during the mixed dentition period, a phase characterized by high biological plasticity. Interceptive correction at this stage may enable early neuromuscular reorganization before compensatory skeletal or muscular patterns are established, ultimately supporting more stable long-term occlusal relationships.

A recent study by Fritz et al.^20^ evaluated the distribution of occlusal forces during the retention phase following orthodontic treatment using the T-Scan system. Their findings revealed that occlusal force distribution remained stable over a 3-month period, regardless of the retention protocol used. This underscores the importance of achieving a balanced occlusion during active treatment, as it likely contributes to long-term stability postintervention. In this context, the improvements in total and segmental force distribution observed in our study suggest that early interceptive correction establishes favorable occlusal conditions that persist beyond appliance removal.

Beyond occlusal balance, this study also demonstrated significant improvements in periodontal health indicators, including reductions in plaque and gingival indices, probing depth, and clinical attachment level. These outcomes support the hypothesis that correcting traumatic occlusion reduces localized inflammatory stimuli, potentially facilitating periodontal healing. In pediatric patients—who often struggle with consistent oral hygiene during the mixed dentition—these improvements are particularly meaningful. Biomechanically, alleviation of excessive occlusal forces may reduce the expression of inflammatory mediators such as prostaglandins and interleukins, thereby promoting gingival and connective tissue recovery^18^.

Comparable findings have been reported by Pellegrino et al.^8^, who observed enhanced occlusal balance and muscle coordination following early crossbite correction using an eruption guidance appliance, and by Almarhoumi and Alwafi^21^, who noted improvements in functional occlusion and periodontal parameters after interceptive treatment. Collectively, these studies reinforce the dual benefit of early orthodontic intervention: correcting malocclusion and promoting periodontal health and overall oral function.

Digital occlusal analysis systems such as T-Scan and OccluSense have become integral to clinical research and orthodontic practice, providing objective assessments of occlusal contact patterns. T-Scan is widely regarded as the gold standard owing to its high temporal resolution, force-mapping capabilities, and validated accuracy in complex occlusal cases^15^. However, its bulkiness, technical complexity, and need for wired sensor placement can limit its usability in pediatric settings, where patient cooperation and chairside efficiency are critical^22^.

In contrast, OccluSense offers a wireless, user-friendly interface and real-time data visualization, making it particularly suitable for interceptive orthodontics in children. Its portability and minimal setup facilitate assessments even in young or anxious patients. Nevertheless, the system provides relatively lower resolution data, and pediatric-specific validation studies remain limited. A systematic review by Velásquez et al.^23^ comprehensively evaluated digital occlusal analysis tools and emphasized that while T-Scan has been consistently validated across multiple clinical contexts, further pediatric-specific research is needed to confirm the reliability of OccluSense. The review also underscored the importance of standardized protocols and calibration methods, particularly in dynamic occlusal environments such as mixed dentition.

From a clinical and biomechanical standpoint, anterior crossbite presents distinct challenges compared with other malocclusions, such as posterior crossbite or Class III skeletal patterns. Posterior crossbite typically involves transverse skeletal discrepancies that often require maxillary expansion, while Class III malocclusion may necessitate orthopedic or surgical interventions due to underlying skeletal imbalances. In contrast, anterior crossbite during the mixed dentition period is frequently of dental origin and can be effectively addressed through interceptive orthodontic approaches such as the Z-spring appliance^21^.

Early correction in such cases not only alleviates functional disturbances and esthetic concerns but also prevents progression to more severe skeletal malocclusions. Timely redirection of eruptive trajectories and normalization of occlusal contacts during growth can guide favorable craniofacial development. As emphasized by the recent expert consensus^24^, interceptive treatment during this developmental window offers a critical opportunity to positively influence long-term occlusal and skeletal outcomes.

Clinically, this study highlights the utility of early interceptive correction in anterior crossbite cases using a Z-spring appliance. By promoting more balanced occlusal load distribution and alleviating excessive focal stress, treatment contributed to enhanced masticatory efficiency and reduced risk of periodontal complications and temporomandibular dysfunction. This finding aligns with that of Turkistani et al.^25^, who emphasized that harmonized occlusal force patterns posttreatment ensure more equal distribution across opposing sides, reducing the biomechanical strain on individual teeth.

Despite the clinically meaningful findings, this study has several limitations. The mixed dentition period is characterized by substantial biological variability, including active tooth eruption, exfoliation, and ongoing craniofacial growth, all of which can influence occlusal dynamics. Although efforts were made to standardize growth patterns and treatment protocols, residual confounding related to developmental changes cannot be fully excluded. Additionally, the absence of a control group limits the ability to differentiate treatment effects from natural maturation. The relatively short follow-up duration restricts the assessment of long-term stability, and certain outcomes, such as total occlusal force, were analyzed descriptively owing to data resolution constraints. The evolution of occlusal force dynamics beyond the initial posttreatment phase remains unknown, warranting extended observation. Finally, the moderate sample size and use of a single measurement tool (OccluSense) may limit the generalizability and reproducibility of the findings.

Future research should prioritize prospective, controlled studies with larger and more diverse pediatric populations. Longitudinal follow-up is essential to evaluate the stability of occlusal force distribution and periodontal outcomes after interceptive treatment. Additionally, comparative validation studies of digital occlusal analysis systems—particularly between OccluSense and T-Scan in children—are warranted to determine reliability across clinical contexts. Establishing standardized protocols, calibration techniques, and uniform outcome measures will be vital for advancing evidence-based practice in pediatric orthodontics and improving the long-term effectiveness of interceptive interventions.

## Conclusions

Interceptive orthodontic treatment of anterior crossbite using a Hawley appliance with a Z-spring yielded measurable improvements in occlusal force distribution and periodontal parameters. The application of computerized occlusal analysis systems facilitated objective evaluation of biomechanical changes, demonstrating a redistribution of occlusal contacts toward more balanced and functional loading patterns. These findings emphasize the therapeutic efficacy of early-phase orthodontic intervention in correcting malocclusion and enhancing periodontal health and occlusal stability.

However, validation of these results through prospective studies incorporating larger sample sizes, appropriate control groups, and extended follow-up durations is warranted to ascertain their long-term stability and clinical significance.

## Supplementary Information

Below is the link to the electronic supplementary material.

Supplementary Material 1

## Data Availability

The datasets used and/or analyzed during the current study are available from the corresponding author on reasonable request.
