# Supplementary material for: Digital occlusal force analysis in anterior crossbite
Source: Sci Rep. 2025 Aug 19;15:30290. doi: 10.1038/s41598-025-16194-z (PMC12365287; doi:10.1038/s41598-025-16194-z)
Supplement: Supplementary file 1 — Supplementary Material 1 [file 41598_2025_16194_MOESM1_ESM.docx]

**Table S1. Summary of Statistical Tests and Comparisons**

| **Statistical Comparison** | **Test Applied** | **Subgroup or Comparison Detail** |
| --- | --- | --- |
| Regional occlusal force (T0 vs T1) | Paired t-test | Pre- and post-treatment (within-subject) |
| Percentage force distribution (T0 vs T1) | Paired t-test | Pre- and post-treatment (within-subject) |
| Periodontal indices (plaque, gingival, CAL, PD) | Wilcoxon signed-rank test | Pre- and post-treatment (within-subject) |
| Percentage force change vs. age, sex, treatment duration | Kruskal–Wallis test | Across age (6–8, 9–11), sex, and treatment duration groups |
| Categorical associations (e.g., gender vs. treatment duration) | Chi-square test | Cross-tabulation of categorical variables |
| Effect size (Cohen’s d) | Effect size calculation | T0 vs T1 comparisons for key force variables |
